# Presence of Viral Genome in Urine and Development of Hematuria and Pathological Changes in Kidneys in Common Marmoset (*Callithrix jacchus*) after Inoculation with Dengue Virus

**DOI:** 10.3390/pathogens2020357

**Published:** 2013-05-13

**Authors:** Meng Ling Moi, Tsutomu Omatsu, Takanori Hirayama, Shinichiro Nakamura, Yuko Katakai, Tomoyuki Yoshida, Akatsuki Saito, Shigeru Tajima, Mikako Ito, Tomohiko Takasaki, Hirofumi Akari, Ichiro Kurane

**Affiliations:** 1 National Institute of Infectious Diseases, 1-23-1 Toyama, Shinjuku-ku, Tokyo, 162-8640, Japan; E-Mails: sherry@nih.go.jp (M.L.M.); tomatsu@cc.tuat.ac.jp (T.O.); hirayama@jata.or.jp (T.H.); stajima@nih.go.jp (S.T.); mito0908@yahoo.co.jp (M.I.); takasaki@nih.go.jp (T.T.); 2 Tokyo University of Agriculture and Technology, 3-5-8 Fuchu, Tokyo, 183-8509, Japan; 3 Japan Anti-tuberculosis Association, 3-1-24 Matsuyama, Kiyose, Tokyo, 204-8533, Japan; 4 Shiga University of Medical Science, Seta Tsukinowa-cho, Otsu, Shiga, 520-2192, Japan; E-Mail: snakamur@belle.shiga-med.ac.jp; 5 National Institute of Biomedical Innovation, 1 Hachimandai, Tsukuba, Ibaraki, 305-0843, Japan; E-Mail: katakai@primate.or.jp; 6 Primate Research Institute, Kyoto University, Inuyama, Aichi, 484-8506, Japan; E-Mails: ytomoyuki@pri.kyoto-u.ac.jp (T.Y.); saito.akatsuki.6a@kyoto-u.ac.jp (A.S.)

**Keywords:** dengue virus, animal model, marmoset, urine, hematuria, kidney

## Abstract

Common marmosets (*Callithrix jacchus*) developed high levels of viremia, clinical signs including fever, weight loss, a decrease in activity and hematuria upon inoculation with dengue virus (DENV). Presence of DENV genome in urine samples and pathological changes in kidneys were examined in the present study. Levels of DENV genome were determined in 228 urine samples from 20 primary DENV-inoculated marmosets and in 56 urine samples from four secondary DENV-inoculated marmosets. DENV genome was detected in 75% (15/20) of marmosets after primary DENV infection. No DENV genome was detected in urine samples from the marmosets with secondary infection with homologous DENV (0%, 0/4). Two marmosets demonstrated hematuria. Pathological analysis of the kidneys demonstrated non-suppressive interstitial nephritis with renal tubular regeneration. DENV antigen-positive cells were detected in kidneys. In human dengue virus infections, some patients present renal symptoms. The results indicate that marmosets recapitulate some aspects of the involvement of kidneys in human DENV infection, and suggest that marmosets are potentially useful for the studies of the pathogenesis of DENV infection, including kidneys.

## 1. Introduction

Dengue fever is a serious global health problem. In dengue fever patients, urine samples contain dengue virus (DENV) genomes and virus antigens were present in renal biopsies. However, the association between disease symptoms from appearance of DENV genome in urine, renal injury (occurrence rate of 2.9–13.3% of dengue patients) and haemolytic uraemic syndrome in the pathogenesis of dengue fever is unclear [1,2]. Poor outcome, including severe dengue and mortality, correlated with poor renal function [1]. To elucidate disease pathogenesis, it is important to establish an animal model that exhibits clinical signs which are comparable to those of human DENV infection. Marmosets develop high levels of viremia and demonstrate changes in biochemical and hematological parameters upon DENV inoculation [3,4]. In the present study, we constantly detected DENV genome in urine samples from DENV-inoculated marmosets. These marmosets also exhibited hematuria and pathological changes in the kidneys. The marmoset DENV infection model appears to recapitulate some aspects of DENV infection, and thus, offer the possibility of use in pathogenesis studies of DENV infection. 

## 2. Results and Discussion

A total of 228 urine samples were obtained on days 1–14 from 20 marmosets after primary infection and 56 urine samples from four marmosets after homologous secondary infection (Table 1). DENV genome was detected in 18 of the 20 primary DENV-inoculated marmosets (Table 1, experiments 1–5). DENV genome was first detected three days after inoculation, and detected up to day 14. The levels of viral genome ranged from 3.8 × 10^3^ to 8.4 × 10^7^ copies/ml. The positive detection rates were 10% (8/82) on days 1–5, 20% (16/79) on days 6–10 and 27% (18/67) on days 11–14 after primary inoculation (Table 2). No DENV genome was detected in 56 urine samples (0%, 0/56) from 4 marmosets re-inoculated with the same serotype (Table 2, experiment 6, marmosets D2-2, D2-3, D2-4 and D2-5) on days 1–14 after inoculation. The detection rates in urine samples were 6% on day 3, the rates ranged from 17 to 31% (mean percentage = 22 ± 4%) on days 4–13, and 35% on day 14. In comparison to detection of viral genome in urine samples, DENV genome was detected on days 2–7 in serum samples (Table 2).

**Table 1:** Levels of dengue viral genome in urine samples from marmosets inoculated with dengue virus (DENV).

Animal ID	Virus strain	Dengue vRNA copy numbers (copies/ml)														
		pfu/ dose	Days after inoculation													
			1	2	3	4	5	6	7	8	9	10	11	12	13	14
**Primary inoculation**																
*Experiment 1*																
D1-1	02-17/1	3.5 × 10^7^	-	NT	NT	NT	7.5 × 10^4^	-	NT	-	-	NT	-	-	-	-
D2-1	DHF0663	6.7 × 10^7^	-	NT	NT	NT	3.8 × 10^3^	2.5 × 10^4^	7.0 × 10^4^	4.0 × 10^4^	5.0 × 10^4^	3.7 × 10^4^	6.5 × 10^4^	4.2 × 10^3^	1.0 × 10^4^	-
D3-1	DSS1403	4.5 × 10^6^	-	NT	NT	NT	-	-	NT	4.8 × 10^4^	-	-	NT	8.5 × 10^4^	-	-
D4-1	05-40/1	1.5 × 10^6^	-	NT	NT	NT	-	-	NT	-	-	NT	-	-	-	-
*Experiment-2*																
D2-2	DHF0663	4.4 × 10^7^	-	NT	8.3 × 10^4^	-	-	-	2.9 × 10^5^	1.8 × 10^5^	-	-	-	8.1 × 10^4^	-	-
D2-3			-	NT	-	7.3 × 10^4^	-	7.1 × 10^4^	1.5 × 10^5^	-	4.8 × 10^5^	-	-	-	-	-
D2-4		1.8 × 10^5^	-	NT	-	-	1.4 × 10^5^	-	-	-	-	-	-	-	-	2.0 × 10^5^
D2-5			-	NT	-	-	-	-	-	-	-	-	-	-	-	-
*Experiment-3*																
D2-6	DHF0663	1.8 × 10^4^	-	-	-	-	-	-	-	-	-	1.0 × 10^5^	-	-	-	-
D2-7			-	-	-	-	-	-	-	-	-	-	-	-	-	8.5 × 10^4^
D2-8		1.8 × 10^3^	-	-	-	-	-	-	-	-	-	-	-	-	-	1.3 × 10^4^
D2-9			-	-	-	-	-	-	-	-	-	-	1.9 × 10^4^	-	-	-
*Experiment-4*																
D2-10	Jam/77/07	1.2 × 10^5^	-	-	-	-	-	-	-	-	-	-	-	3.0 × 10^5^	-	-
D2-11			-	-	-	6.7 × 10^3^	-	-	-	-	-	-	-	-	-	4.2 × 10^5^
D2-12	Mal/77/08	1.9 × 10^5^	-	-	-	-	-	-	-	NT	-	5.9 × 10^6^	1.7 × 10^5^	-	7.8 × 10^5^	-
D2-13			-	-	-	-	-	-	-	-	1.3 × 10^6^	-	4.4 × 10^5^	-	-	8.1 × 10^5^
*Experiment-5*																
D2-14	DHF0663	6.7 × 10^7^	-	-	-	*	*	*	*	*	*	*	*	*	*	*
D2-15			-	-	-	-	-	*	*	*	*	*	*	*	*	*
D2-16			-	-	-	-	-	-	*	*	*	*	*	*	*	*
D2-17			-	-	-	2.7 × 10^5^	2.6 × 10^5^	4.4 × 10^6^	-	-	NT	8.4 × 10^7^	5.9 × 10^7^	-	1.9 × 10^7^	8.2 × 10^6^

- indicates viral RNA below limit of detection using RT-PCR, NT indicates not tested, * indicates that the marmosets were sacrificed and no samples were collected.

**Table 2:** Positive rates in the detection of DENV genome in urine samples on each of the 14 days following inoculation with DENV, in comparison with the previously reported data of serum samples [3].

Days after inoculation	Number of serum samples positive by RT-PCR (% positive, total tested)	
	Urine	Sera *
Day 1	0/20 (0)	NT ^†^
Day 2	0/12 (0)	12/12
Day 3	1/16 (6)	7/8
Day 4	3/15 (20)	11/11
Day 5	4/19 (21)	4/5
**Days 1–5**	**8/82 (10)**	**34/36 (94)**
Day 6	3/18 (17)	NT
Day 7	3/14 (21)	11/18 (61)
Day 8	3/16 (19)	0/1 (0)
Day 9	3/16 (19)	NT
Day 10	4/15 (27)	0/4 (0)
**Days 6–10**	**16/79 (20)**	**11/23 (48)**
Day 11	5/16 (31)	NT
Day 12	4/17 (24)	NT
Day 13	3/17 (18)	NT
Day 14	6/17 (35)	0/17 (0)
**Day 11–14**	**18/67 (27)**	**0/17 (0)**
**Total**	**42/228 (18)**	**45/76 (59)**

* The DENV genome levels in serum samples were previously reported [3], ^†^ NT: indicates test not done.

In human dengue patients, DENV genome was detected in 30% (9/30) of urine samples as compared to 79% (34/43) in serum samples from days 1–5 after onset of disease, and 61% of urine samples as compared to serum samples (11%) on days 10–16 after onset of disease [5]. In marmosets, the rate of DENV genome-positive urine samples (10%, 8/82, Table 2) was lower as compared those of serum samples (94%, 34/36) on days 1–5 after DENV inoculation, and DENV genome was detected at a higher rate in urine samples (27%, 18/67) as compared to serum samples (0%, 0/17) on days 11–14 after infection (Table 2) [3]. Thus, the kinetics of viral genome clearance in urine and serum samples in marmosets were similar to those of human DENV patients. It is of interest that for both DENV patients and DENV-inoculated marmosets, infectious DENV was not detected in urine samples even when DENV genome was detected [5]. Limitations of our study include lack of data on the presence or absence of infectious virus in kidneys. Although DENV genome and viral antigen were present in kidneys, the antigens could represent reabsorbed immune complexes after clearance, and may suggest mechanisms other than viral replication in renal tissue, are involved in renal dysfunction during DENV infection. 

**Figure 1:**
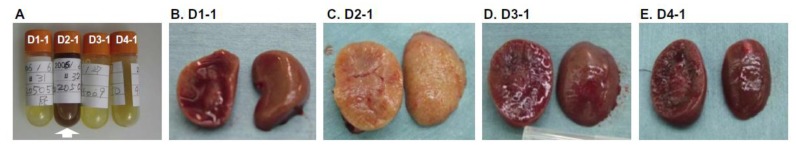
Gross appearance of kidney and urine specimens of DENV inoculated marmosets. Urine samples for D1-1, D3-1 and D4-1 were pale yellow and clear (Figure 1A). Hematuria was detected in urine from DENV-2 inoculated marmoset D2-1 (indicated by a white arrow). Gross appearance of kidneys from marmosets inoculated with DENV-1 (marmoset D1-1, Figure 1B), DENV-2 (marmoset D2-1, Figure 1C), DENV-3 (marmoset D3-1, Figure 1D) and DENV-4 (marmoset D4-1, Figure 1E). The kidney from DENV-2 inoculated marmoset (D2-1) was swollen and fawn-colored (Figure 1C).

In comparison to the clear appearance of urine samples from DENV-1, DENV-3 and DENV-4 inoculated marmosets (D1-1, D3-1 and D4-1), marmoset D2-1 demonstrated apparent hematuria (Figure 1A). Microscopic hematuria was detected in marmoset D2-17 on days 5 and 8 after DENV inoculation but was not detected in other 7 marmosets tested (D2-6, D2-7, D2-8, D2-9, D2-14, D2-15, and D2-16) on days 1 to 14. Marmosets D1-1, D2-1 and D4-1 exhibited signs of ascitis formation (data not shown). Kidneys of both sides from marmoset D2-1 were swollen and fawn-colored (Figure 1C).

**Figure 2:**
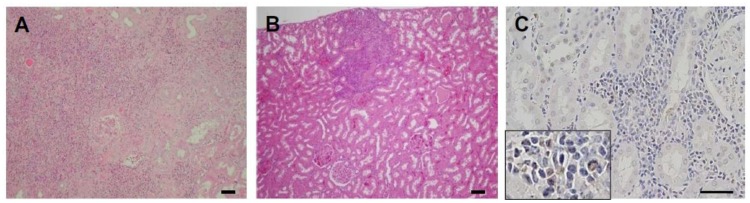
Histopathology associated with DENV-2 inoculation in marmosets. Severe non-suppressive interstitial nephritis with renal tubular degeneration was detected in kidney of DENV-2 inoculated marmoset (marmoset D2-1, Figure 2A). DENV-2 inoculated marmosets also exhibited moderate interstitial nephritis, and segmental glomerulosclerosis and renal tubular atrophy (D2-1, Figure 2B). Dengue viral antigen was detected in cells that morphologically correspond to lymphocytes and macrophages in the kidney from marmoset D2-17 (Figure 2C). Scale bar = 50 µm.

Upon histopathological examination, the kidney from marmoset D2-1 demonstrated significant renal lesions (Figure 2A,B). Despite the limited numbers of marmosets evaluated, levels of DENV genome in urine samples of two marmosets (D2-1 and D2-17) which demonstrated hematuria and significant renal lesions (mean virus titer = 3.7 log10 copies/ml) were higher as compared to urine samples from 18 marmosets which did not demonstrate hematuria (mean virus titer = 0.5 log10 copies/mL, P < 0.01; two-tailed Student’s t-test). Mean genome positive days of urine samples for marmosets D2-1 and D2-17 was 8.0 days as compared to 1.4 days for marmosets that did not demonstrate hematuria. 

Renal dysfunction, occurs in 2.9–13.3% of human dengue patients, and is associated with disease severity [1,2]. Severity of renal dysfunction was also associated with higher percentages of severe dengue and mortality in humans. Although the number of marmosets used in the present study was limited, two DENV-inoculated marmosets (2/18, 11%) demonstrated clinical signs of the renal system. In addition to viremia and biochemical changes, development of these clinical signs in marmosets, suggests the feasibility of marmosets as an animal model to elucidate the pathogenesis of DENV infection, including the renal system.

## 3. Experimental Section

A total of 20 male common marmosets (*Callithrix jacchus*) were used in accordance with “Guides for animal experiments according to institutional guidelines (Approval no. 608011, 609014 and Approval no. 20-003, 21-013). The marmosets were inoculated subcutaneously in the back with either DENV-1, DENV-2, DENV-3 or DENV-4. Four marmosets (D2-2, D2-3, D2-4 and D2-5) were inoculated with the same serotype (DENV-2) at 33 weeks after primary inoculation [3]. DENV type 1 (DENV-1), 02-17/1 strain, DENV-2 DHF0663 strain (Accession no. AB189122), DENV-2 Jam/77/07 strain, DENV-2 Mal/77/08 strain, DENV-3 DSS1403 strain (Accession no. AB189125), and DENV-4 05-40/1 strain, were used for inoculation studies. Day 0 was defined as the day of virus inoculation. Urine samples were examined for gross hematuria for all 20 marmosets and a urine dipstick (Bayer Urine Dipstick, IN, USA) for marmosets D2-6, D2-7, D2-8, D2-9, D2-14, D2-15, D2-16 and D2-17). For histological analyses, paraffin-embedded tissues sections (4µm sections) were deparaffinized and stained with HE stain. For immunohistochemical analyses, sections were stained using HRP-conjugated marmoset polyclonal anti-DENV antibody. For viral RNA isolation, High Pure Viral RNA Kit (Roche Diagnostics) was used. To determine the levels of dengue viral RNA, each sample was assayed in a 25 µL reaction containing 5 µL of sample RNA, 0.9 µM of each forward and reverse DENV-serotype-specific primer, RT/RNAse Inhibitor Mix, 0.2 µM TaqMan DENV-serotype-specific probe, and TaqMan Universal Master Mix (Invitrogen). The thermal conditions were (1) reverse transcription at 48 °C for 30 minutes, (2) *Taq* polymerase activation at 95 °C for 10 minutes, (3) forty cycles of PCR consisting of denaturing at 95 °C for 15 seconds and annealing at 57 °C for 1 min [3]. RNA standards with RNA copies of 10^8^ to 10^4^ were used to quantify the dengue viral RNA. All TaqMan RT-PCR assays were performed in duplicate. Replicate variability threshold was set at 0.5, the RT-PCR detection limit of this study ranges from 3.1 × 10^2^ to 4.4 × 10^4^ copies/ ml for four DENV serotypes. Data analysis was done using Microsoft Excel and GraphPad Prism Statistical Package (Graphpad Software, CA, USA). Percentage of genome positive days was calculated using the formula (number of days with positive viral genome detection/ number of sampling days) × 100%. 

## 4. Conclusions

Common marmosets demonstrate DENV genome in urine, hematuria and pathological changes of the kidneys upon DENV infection. The recapitulation of these clinical aspects of DENV infection, including the involvement of kidneys, suggests the feasibility of the use of marmosets for studies on the pathogenesis of DENV infection.
